# Consequences of gene editing of *PRLR* on thermotolerance, growth, and male reproduction in cattle

**DOI:** 10.1096/fba.2024-00029

**Published:** 2024-06-18

**Authors:** Camila J. Cuellar, Thiago F. Amaral, Paula Rodriguez‐Villamil, F. Ongaratto, D. Onan Martinez, Rémi Labrecque, João D. de Agostini Losano, Eliab Estrada‐Cortés, Jonathan R. Bostrom, Kyra Martins, D. Owen Rae, Jeremy Block, Quinn A. Hoorn, Bradford W. Daigneault, Jonathan Merriam, Michael Lohuis, Serdal Dikmen, João H. J. Bittar, Tatiane S. Maia, Daniel F. Carlson, Sabreena Larson, Tad S. Sonstegard, Peter J. Hansen

**Affiliations:** ^1^ Department of Animal Sciences University of Florida Gainesville Florida USA; ^2^ Acceligen Eagan Minnesota USA; ^3^ Department of Large Animal Clinical Sciences, College of Veterinary Medicine University of Florida Gainesville Florida USA; ^4^ The Semex Alliance Guelph Ontario Canada; ^5^ Campo Experimental Centro Altos de Jalisco Instituto Nacional de Investigaciones Forestales, Agrícola y Pecuarias Tepatitlán de Morelos Mexico; ^6^ Department of Animal Science University of Wyoming Laramie Wyoming USA; ^7^ Faculty of Veterinary Medicine, Department of Animal Science Bursa Uludag University Bursa Turkey; ^8^ Recombinetics, Inc Eagan Minnesota USA; ^9^ Present address: Genus PLC/ABS São Paulo Brazil; ^10^ Present address: Genus plc De Forest Wisconsin USA; ^11^ Present address: University of Wisconsin Biotechnology Center Madison Wisconsin USA

**Keywords:** cattle, gene editing, PRLR, slick allele, thermotolerance

## Abstract

Global warming is a major challenge to the sustainable and humane production of food because of the increased risk of livestock to heat stress. Here, the example of the prolactin receptor (*PRLR*) gene is used to demonstrate how gene editing can increase the resistance of cattle to heat stress by the introduction of mutations conferring thermotolerance. Several cattle populations in South and Central America possess natural mutations in *PRLR* that result in affected animals having short hair and being thermotolerant. CRISPR/Cas9 technology was used to introduce variants of *PRLR* in two thermosensitive breeds of cattle – Angus and Jersey. Gene‐edited animals exhibited superior ability to regulate vaginal temperature (heifers) and rectal temperature (bulls) compared to animals that were not gene‐edited. Moreover, gene‐edited animals exhibited superior growth characteristics and had larger scrotal circumference. There was no evidence for deleterious effects of the mutation on carcass characteristics or male reproductive function. These results indicate the potential for reducing heat stress in relevant environments to enhance cattle productivity.

## Graphical abstract

Consequences of gene editing of PRLR on thermotolerance, growth, and reproduction in cattle. Camila J. Cuellar*, Thiago F. Amaral, Paula Rodriguez‐Villamil, F. Ongaratto, D. Onan Martinez, Rémi Labrecque, João D. de Agostini Losano, Eliab Estrada‐Cortés, Jonathan R. Bostrom, Kyra Martins, D. Owen Rae, Jeremy Block, Quinn A. Hoorn, Bradford W. Daigneault, Jonathan Merriam, Michael Lohuis, Serdal Dikmen, João H.J. Bittar, Tatiane S. Maia, Daniel F. Carlson, Sabreena Larson, Tad S. Sonstegard, and Peter J. Hansen. CRISPR/Cas9 technology was used to introduce variants of *PRLR* in two thermosensitive breeds of cattle – Angus and Jersey. Gene‐edited animals exhibited superior ability to regulate vaginal temperature (heifers) and rectal temperature (bulls) compared to animals that were not gene‐edited and superior growth characteristics. Figure created with Biorender.com

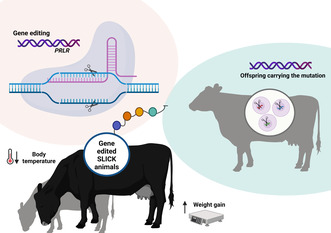

## 1 INTRODUCTION

One of the major challenges to the sustainable and humane production of food from animal sources is global warming.^1^, ^2^ Many species used for food production are homeotherms that regulate core body temperature within a narrow range. Environmental conditions such as high air temperatures, solar radiation, and high humidity cause heat stress which exacerbates thermoregulation in homeotherms. The resultant changes in physiology can compromise reproduction, production, and health. In cattle, for example, heat stress can reduce fertility in both sexes, impair fetal development, lower postnatal growth, reduce milk yield, and cause death.^2^, ^3^ Other impacts of global climate change on animal agriculture include changes in rainfall and the production of crops and grasses.^4^

Evolutionary forces acting on Bovini have generated extensive genetic diversity including adaptation to climatic conditions.^5^, ^6^ Many modern breeds of cattle that originated in or adapted to hot regions of the world have been shown to exhibit a superior ability to regulate body temperature during heat stress.^3^ Introduction of specific allelic variants of genes responsible for resistance to heat stress in thermotolerant cattle into those more sensitive to heat stress could be a useful strategy for minimizing the consequences of heat stress. Gene editing is a superior approach for doing so as compared to conventional gene introgression strategies based on crossbreeding because only the gene underlying the trait of interest is modified, and the process can be completed in a single generation without loss of efficiency for animal production.

To date, the only gene in cattle shown to possess mutations conferring thermotolerance is prolactin receptor (*PRLR*).^7^, ^8^, ^9^ In particular, a total of six separate single nucleotide polymorphisms (SNP) have been identified in cattle breeds that arose in the Caribbean Basin to cause a premature stop codon and result in a predicted protein in which the C‐terminus containing critical residues for JAK–STAT signaling are absent. Collectively, these mutations are called slick mutations because animals inheriting either one or two copies of the mutation exhibit a sleek, short hair coat.^7^, ^10^, ^11^ One function of prolactin is to inhibit hair growth^7^ so slick cattle function as if signaling by prolactin is enhanced, at least concerning hair growth. Cattle inheriting one slick allele of *PRLR* exhibited superior ability to regulate body temperature and produce milk during heat stress.^10^, ^11^, ^12^, ^13^

Here we demonstrate the use of gene editing techniques to introduce novel variants into *PRLR* that result in cattle with a slick phenotype. The variants were introduced into cattle of two breeds originating from Northern Europe that are both sensitive to heat stress and important globally for beef (Angus) or dairy production (Jersey). Results demonstrated that successfully edited animals were phenotypically slick in appearance and exhibited a superior ability to regulate body temperature as compared to animals that were not gene‐edited. Moreover, gene‐edited animals exhibited superior growth characteristics through the first year of life, which indicates the potential for *PRLR* truncation mutations to enhance cattle productivity in tropical and subtropical zones of production. Prolactin is a hormone with multiple target tissues including the male and female reproductive tract,^14^, ^15^ yet there was no evidence of deleterious effects of the slick mutation on reproductive function in males.

## 2 MATERIALS AND METHODS

### 2.1 Overview of experimental procedures

Procedures for use of animals was approved by the University of Florida Institutional Animal Care and Use Committee (project IACUC202200000747). Angus and Jersey calves derived from embryos subjected to gene editing using CRISPR/Cas9 technology were born during the period from July to October 2021. Animals were suckled by their dams until weaning on March 15, 2022. Calves and their dams were maintained together on bermudagrass and bahiagrass pastures at the University of Florida Boston Farm—Santa Fe River Ranch Beef Unit at Bland, Florida (29°55’ N 82°28’ W). After weaning, all animals were maintained together for the remainder of the experiment at the University of Florida Beef Teaching Unit at Gainesville Florida (29°37’ N 82°21’ W), except for Jersey heifers. Since there were no non‐edited Jersey heifers, this subgroup was excluded from all measurements. Animals were fed bermudagrass hay ad libitum and twice‐daily corn gluten meal‐based supplement for growing beef animals whose composition varied with age.

Measurements of body weight were collected at birth using an electronic scale. Other measurements were determined at fixed times so that animals varied in age by 73 days at the time of measurement. Body weight and plasma concentrations of prolactin and insulin‐like growth factor 1 (IGF1) were measured at weaning on March 15, 2022, when animals had an average age of 221 days (range 178–254 days). Body temperature measurements were obtained in the summer during heat stress. Vaginal temperature in Angus heifers was measured twice, from August 15 to 19 of 2022 (average age = 411 days) and from August 21 to 25 of 2022 and (average age = 417 days). Body weight was again measured by an electronic scale on August 15 (average age = 374 days). Rectal temperature, skin temperature, and respiration rate of all animals were assessed on four occasions (August 19, 23, 25, and 30, 2022) and hair weight was assessed once (August 15, 2022). Scrotal circumference was measured in bulls on August 19, 2022 (average age = 378 days) and November 29, 2022 (average age = 480 days). Body weight (by electronic scale) and carcass characteristics estimated by ultrasound were determined on November 18, 2022 (average age = 469 days). Semen was collected from bulls by electroejaculation on November 10, 17, and 29, 2022, and December 8 and 22, 2022 [range in average ages = 461 (November 10) to 503 days (December 22)]. Semen was analyzed for sperm kinematics using computer‐assisted sperm analysis. Subsequently, the fertilizing ability of frozen–thawed semen was tested using in vitro fertilization. Finally, a second blood sample to measure plasma concentrations of prolactin and IGF1 was obtained on January 23, 2023 (average age = 535 days).

### 2.2 | Generation of gene‐edited calves

Cumulus oocyte complexes (COC) [*n* = 986 oocytes, *n* = 9 replicates; four rounds for Angus (590 COC) and 5 rounds for Jersey (346 COC)] were obtained by transvaginal ultrasound‐guided follicular aspiration from seven different Angus female donors and four different Jersey donors. The COC were placed into a maturation medium and shipped overnight at 38.5°C to Acceligen. After maturation for 20–24 h at 38.5°C, COC from each donor were washed and separately distributed into groups of up to 10–15 COC in 50 μL drops of prewarmed fertilization medium called Fert‐TALP^16^ covered by mineral oil. Frozen–thawed semen from three Angus bulls and two Jersey bulls was used. For each donor, sperm from a single bull was used. After thawing, spermatozoa were selected for fertilization using BoviPure® (Nidacon, Healdsburg, CA, USA). Sperm were added to each drop to a final concentration of 1 × 10^6^/ mL and incubated at 38.8°C with 5.5%–6.5% CO_2_ and atmospheric oxygen. After 12–18 h, presumptive zygotes were denuded manually.

After removal of the zona pellucida, presumptive zygotes were microinjected 24 h after fertilization through intracytoplasmic microinjection with 10–15 pL of gene editing reagents. The proprietary synthetic guide RNA (sgRNA) was designed to cut prior to the critical residues for JAK–STAT signaling. The gene editing reagents were combined in injection buffer (5 mM Tris–HCl, 100 μM ethylenediamine tetraacetic acid) to a final concentration of 25 ng/μL Alt‐R™ S.p. HiFi Cas9 Nuclease V3 (IDT, Coralville IA, USA) and 25 ng/μl PRLR c.9.6 sgRNA (Synthego, Redwood City, CA, USA). Injected zygotes were distributed into 100 μL drops (15–20 each) of culture medium (BO‐IVC, IVF Bioscience, Falmouth, UK) covered in mineral oil and incubated at 38.8°C with 5% CO_2_ and 5% O_2_ for 7 days. Grade 1 blastocysts^17^ were vitrified in Cryolock straws (FUJIFILM Irvine Scientific, Santa Ana, CA, USA) in a solution of 20% (v/v) dimethyl sulfoxide and 20% (v/v) ethylene glycol.

For Angus heifers, the overall cleavage rate was 67% and percent of oocytes becoming blastocysts was 31%. For Jersey heifers, the overall cleavage rate was 53% and percent of oocytes becoming blastocysts was 16%. Vitrified blastocysts were warmed, washed, and transferred to non‐pregnant crossbred beef cows of various admixtures of *Bos taurus* and *B. indicus* genetics. Blastocysts were loaded individually into 0.25 mL embryo transfer straws in HEPES‐TALP medium^18^ containing 10% (v/v) fetal bovine serum and 50 μM dithithreitol and transported to the farm in a portable incubator at 38.5°C for transfer into recipients. Ovulation of the recipients was synchronized as follows. Each female received an intravaginal progesterone‐releasing device (1.38 g progesterone, EAZI‐BREED CIDR, Zoetis, Kalamazoo, MI, USA) on day 0 of the procedure and was injected, i.m., with 100 μg gonadotropin‐releasing hormone (GnRH) (Factrel®, Zoetis). Seven days later, the CIDR was removed and 25 mg prostaglandin F2a (Lutalyse®, Zoetis) was administered, i.m. Another injection of 100 μg GnRH was administered 72 h later to induce ovulation (i.e., day 0 of anticipated ovulation). Ovaries were examined by transrectal ultrasonography to determine the presence of a corpus luteum at day 6 after expected ovulation. On day 7, each cow with a corpus luteum received a single embryo transferred to the uterine horn ipsilateral to the ovary bearing a corpus luteum. Pregnancy diagnosis was performed on days 30 and 60 of gestation by transrectal ultrasonography. Calving data were recorded.

### 2.3 Verification of gene editing

Genome DNA was isolated from ear punch tissue using either the Puregene Tissue Kit (Qiagen) or the DNeasy Blood and Tissue kit (Qiagen) according to the manufacturer's instructions. Next‐generation sequencing (NGS) was used to determine allele composition and percentage. Briefly, the target region was amplified to generate a 477 bp PCR product. PCR products were purified with the QIAquick PCR Purification kit (Qiagen) and submitted to Genewiz for Amp‐EZ NGS according to their instructions. Data were analyzed with Geneious Prime (www.geneious.com) using the Analyze CRISPR Editing Results tool. Acceptance criteria for animals considered to have bi‐allelic “slick” edits were animals with indels predicted to remove the critical residues for JAK–STAT signaling.

The NGS approach is limited by the small sized amplicon. Thus we performed two additional PCR assays to rule out large deletions missed by NGS. PCR was conducted with primers to generate an 1146 bp product [SLICK Long distance (LD) F1 5’‐CGAGCTCTGGAAAGCCAAGA‐3′ and SLICK Long R1 5’‐AGCTGTTGGACAAAGAGGCA‐3′] and 2131 bp product (*PRLR* LD F1 5’‐CATCCTCCCACCAGTTCCAG‐3′ and *PRLR* LD R1 5’‐AGTGCATAAAACCTGCGGGA‐3′). Amplicons were resolved by agarose electrophoresis to visualize deviations from the full length. No additional deletions were identified by these assays that had not already been identified by NGS.

### 2.4 Vaginal temperature

Vaginal temperature was measured using an iButton 1922 L (Maxim Integrated, San Jose, California, USA) as previously described.^11^ Each iButton was set to measure in 15 min intervals for 5 days and set to 0.0625°C accuracy and placed inside of a blank (i.e., no progesterone) EASY‐BREED CIDR device (Zoetis, Kalamazoo, MI, USA). The iButton‐CIDR assembly was placed in the vagina for 5 days, with recording from 1630 H on day 0 until 1600 H on day 5. Animals moved freely in their pastures during data recording. The procedure was performed on two occasions.

Weather data were obtained from Alachua County, Florida for the month of August 2022 by downloading from https://fawn.ifas.ufl.edu/. Average daily dry bulb temperature was obtained from the dry bulb temperature recorded 2 m from the ground and average daily relative humidity was used to calculate THI, using the following equation^19^:

$$\mathrm{THI}=\left(1.8\;x\;\mathrm{Tdb}+32\right)–\left[\left(0.55–0.0055\;x\;\mathrm{RH}\right)\;x\;\left(1.8\;x\;\mathrm{Tdb}–26.8\right)\right].$$

where Tdb = dry bulb temperature (°C) and RH = relative humidity.

### 2.5 Rectal temperature, skin temperature, and respiration rate

Measurements were made while the animals were in a holding chute. The first animal was measured at 1500 H and the last measurement was recorded at 1630 H. Rectal temperature was measured using a digital GLA M750 thermometer (GLA Agricultural Electronics, San Louis Obispo, CA, USA). The thermometer was inserted approximately 7 cm into the rectum, and it was read after 1 min of equilibration. At the same time, respiration rate (by counting flank movements) and skin temperature at two locations on the right side of the animal (neck and loin) were recorded. Skin temperature was measured using an infrared thermometer (Sixth Sense LT300 Infrared Thermometer, TTI instruments, Williston, VT, USA) while holding the device ~15 mm from the skin surface.

### 2.6 Hair weight

Hair growth was measured by shaving a 7.5 cm × 5 cm area between the hook and the pin bones of the pelvis on the right side of the animal. Shaved hair was placed in a pre‐weighed plastic bag.

### 2.7 Carcass traits

Carcass ultrasound was performed as previously described.^20^ The right side of the animal was used for carcass ultrasound. Measurements included the cross‐sectional area of the *longissimus thoracis* muscle between the 12th and 13th ribs (ribeye area) and subcutaneous fat thickness as well as the intramuscular fat percentage through a longitudinal section collected at the 11th, 12th, and 13th ribs and subcutaneous fat thickness at the same locations. An additional measurement of subcutaneous fat thickness was measured at the intersection of the *biceps femoris* and *gluteus medius* muscles.

### 2.8 Scrotal measurements

Scrotal circumference was measured using a scrotal tape. Testicles and the overlying scrotal skin were manually pulled to the bottom of the scrotum and scrotal skin was compressed before pulling the tape tight and recording the circumference.

### 2.9 Semen collection

Semen was collected on different occasions from each bull using electroejaculation. After measuring sperm concentration with a hemocytometer, semen was extended by dilution with a warmed (38.5°C) egg yolk citrate extender (Southeastern Semen Services, Wellborn, FL, USA). Semen was diluted either 1:1 or 1:2 (v/v) depending on sperm concentration. Extended semen was then placed in 0.5 mL straws and cryopreserved.

### 2.10 Computer‐assisted semen analysis

At the time of semen collection, a homogenous sample of 200 μL ejaculate was diluted with an equal volume (1:1) in modified non‐capacitating Tyrode albumin lactate pyruvate medium free of bicarbonate and albumin (35 mM HEPES, pH 7.3, 1 mM CaCl_2_, 4 mM KCl, 130 mM NaCl, 5 mg/mL polyvinylpyrrolidone, 1.3 mM sodium pyruvate, 14 mM fructose, 89 μM penicillin, 0.14 μM streptomycin). Samples were stored in a water bath at 24°C and immediately transported to the laboratory for analysis by computer‐assisted semen analysis (CASA‐IVOS II System, Hamilton Thorne, Beverly, MA, USA). Prior to analysis, the sperm samples were warmed at 37°C and the kinematics assessment was conducted as previously described.^21^

### 2.11 Capacity of sperm to fertilize oocytes and generate blastocysts

Frozen–thawed, extended sperm from slick and non‐slick bulls were used to fertilize oocytes and the resultant embryos were cultured until the blastocyst stage. Oocytes were harvested from ovaries collected at a local abattoir. Each bull was tested separately in 3 independent fertilization procedures using ~60 oocytes/bull for fertilization per procedure. The final concentration of sperm for each bull in the fertilization drops was 1 × 10^6^/mL. Conditions for harvesting oocytes, fertilization, and embryo culture were performed as previously described^18^ except that the culture medium for embryos was BO‐IVC (IVF Biosciences). Cleavage of putative zygotes (i.e., oocytes exposed to spermatozoa) was assessed on day 3 after fertilization. The number of blastocysts was recorded on day 7.5.

### 2.12 Blood samples

Blood was collected by coccygeal vessel puncture into a tube containing ethylenediamine triacetate (BD Bioscience, East Rutherford, NJ, USA). Blood was placed on ice until centrifugation at 2000 × g at room temperature for 30 min. Plasma was harvested and stored at −80°C.

### 2.13 | Hormone assays

Concentrations of prolactin were measured by enzyme‐linked immunoassay using a commercial kit (catalog number SEA846Bo, Cloud‐Clone Corp., Katy, TX, USA) while following the manufacturer's protocol. The assay has been previously validated.^22^ The intra‐assay coefficient of variation was 5.6%. Concentrations of IGF1 were measured by enzyme‐linked immunoassay (SG100; R&D Systems, Inc., Minneapolis, MN, USA). The assay exhibits 100% cross‐reactivity with bovine IGF1 and has been validated.^23^ The intra‐assay coefficient of variation was 3.5%.

### 2.14 | Statistical analysis

Initial statistical analyses indicated that there were no significant differences between non‐edited animals and mosaic animals for any trait measured. For statistical purposes, therefore, slick animals were considered as those in which there was biallelic editing of *PRLR* and non‐slick animals were those in which there was either no edit or where animals were mosaic. Data were analyzed using procedures from the SAS statistical package (SAS v 9.4, Cary, NC, USA). Continuous data were analyzed by analysis of variance using either the GLM procedure or, for repeated measures (body temperatures, semen analysis), GLIMMIX. Categorical data (i.e., results of in vitro fertilization where each oocyte was the experimental unit) were analyzed by logistic regression using GLIMMIX. For repeated measures designs, the animal was considered random and other main effects were considered fixed. Body weight exhibited heterogeneity of variance and were log‐transformed before analysis. Body weight data are reported as means of untransformed data. Depending on the structure of the data, statistical models included genotype (slick vs non‐slick), sire, dam, sex, time, replicate, interactions, and age at the time of measurement as a covariate. Data were reanalyzed after removing terms in the model where the *F* value was <1.0. Statistical effects for genotype and interactions with genotype where *p* was <0.10 or less are reported.

## 3 RESULTS

### 3.1 Gene editing

**FIGURE 1:**
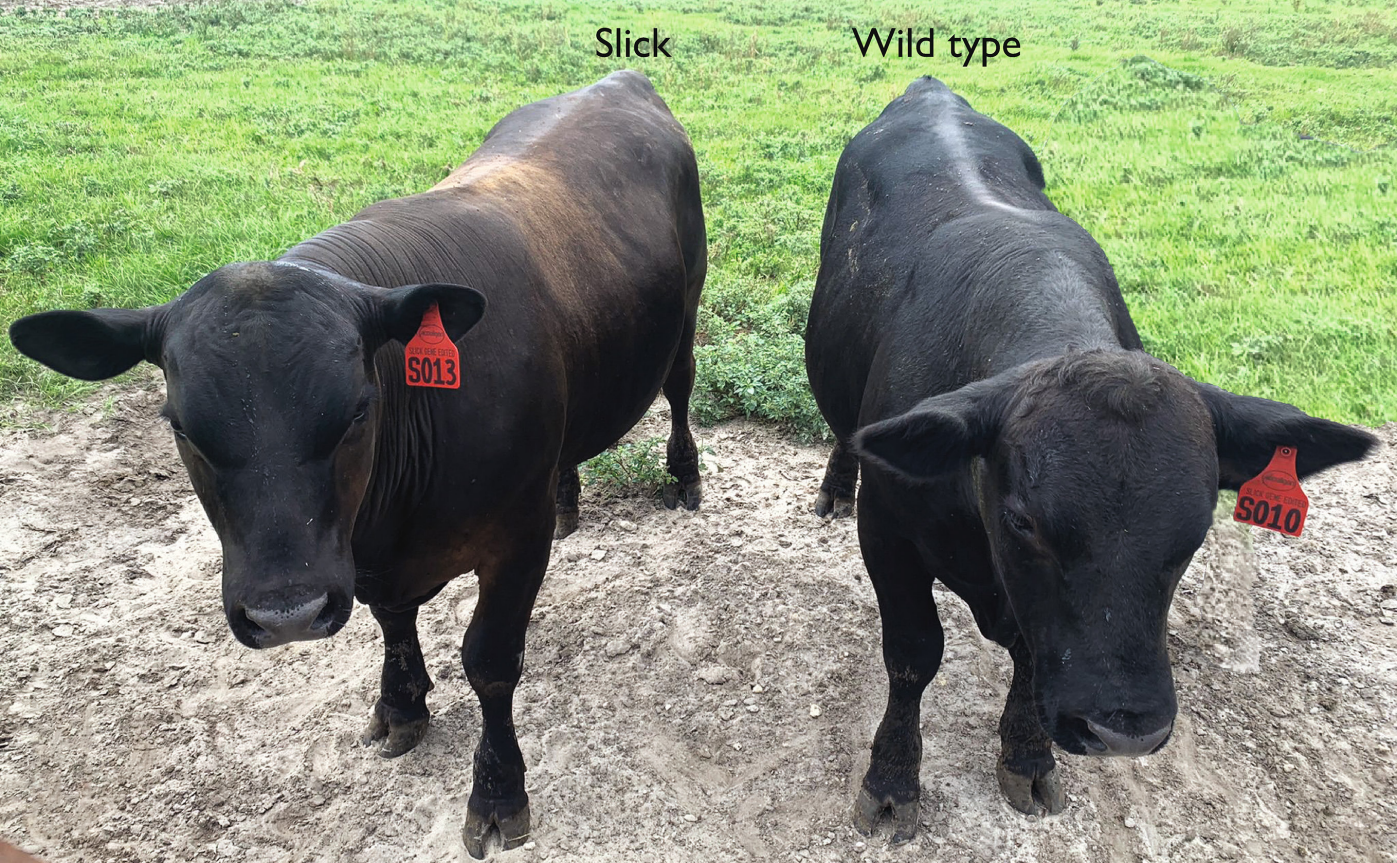
Example of differences in phenotype between slick and non‐slick animals. Shown are two Angus bulls. The photograph was taken on August 30, 2022, when animals were 346–422 days old and when animals had short, summer hair coat. Partial image of a third animal to the right of the image has been digitally removed. The animal inheriting the edited version of *PRLR* (ear tag S013) has a very short hair coat typical of slick animals. This is most easily visualized in the photograph by comparing appearance of the slick animal with the wild‐type animal with ear tag S010. Differences in hair length are most visually noticeable in the region of the face (notice that hair in the poll is largely absent in the slick animal and its facial hair is shorter) and in the neck (notice the appearance of wrinkles in the slick animals which are visible because of the very short hair coat).

A total of 15 Angus (six bulls and nine heifers) and six Jersey (three bulls and three heifers) calves were born following embryo transfer (Table S1). Of these, 6 Angus and 5 Jersey were successfully bi‐allelically edited and expected to disrupt critical JAK–STAT signaling (i.e., 11/21 or 52%) (Table S2). Another 3 Angus were mosaic with the degree of editing varying from 4%–29% (Table S2). A total of five Angus and one Jersey were wild‐type with no editing of *PRLR*. An example of the appearance of slick and wild‐type animals are shown in Figure 1. Phenotypically, mosaic animals appeared similar in hair length to wild‐type animals. In addition, measurements of the wild‐type animals were not statistically different from the mosaic animals. Therefore, subsequent statistical analysis was performed by comparing gene‐edited animals (termed slick) with non‐slick animals (wild‐type and mosaic animals). Jersey females were excluded from analyses because all animals were gene‐edited. After excluding an injured Jersey bull that was euthanized, an Angus heifer that was stillborn, and the Jersey heifers (because all were gene‐edited and there would be no corresponding control), the number of slick animals subjected to statistical analysis was four Angus heifers, two Angus bulls and one Jersey bull. The number of non‐slick animals was four Angus heifers, four Angus bulls, and one Jersey bull.

### 3.2 Body temperature

**FIGURE 2:**
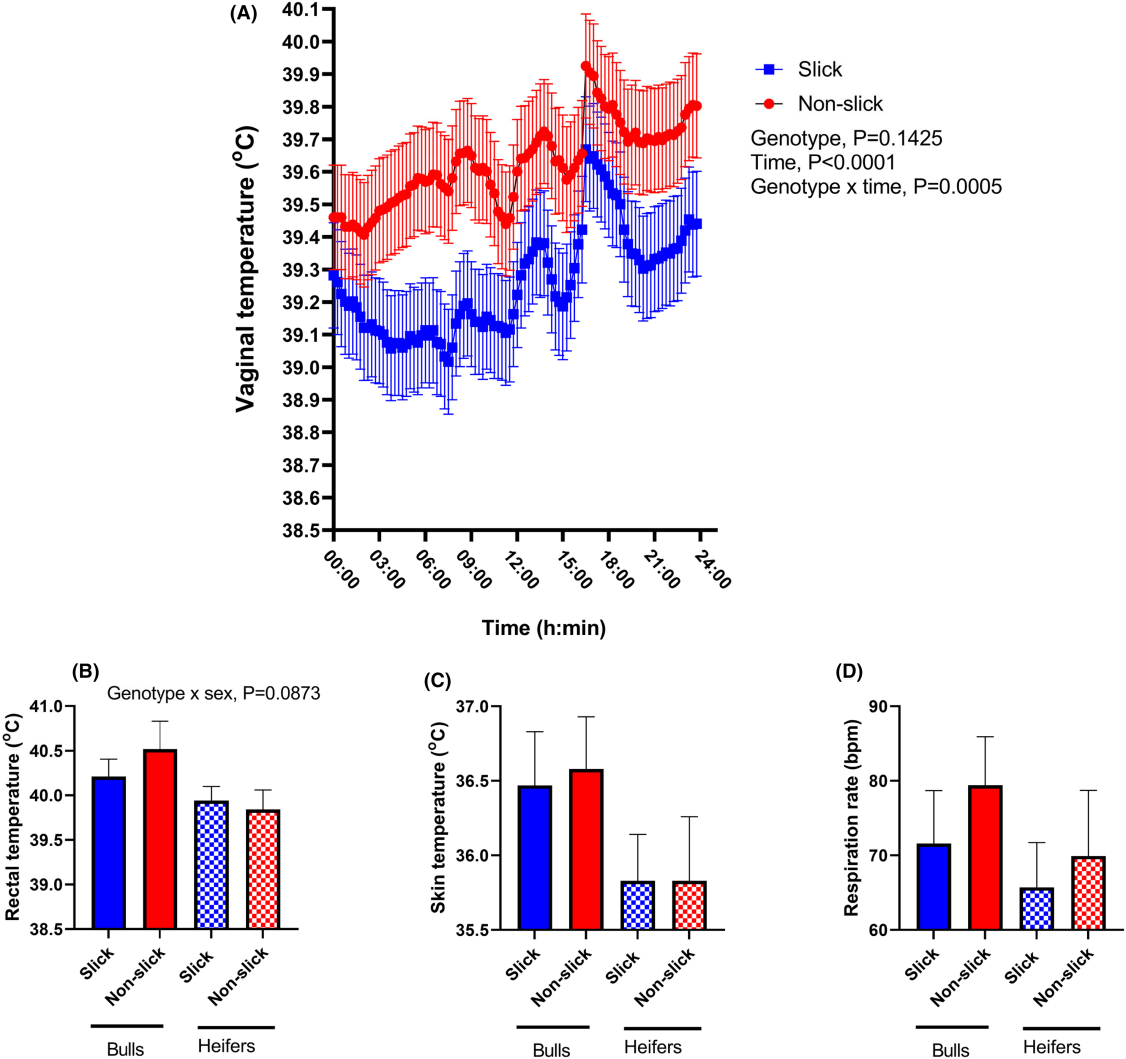
Differences between slick and non‐slick animals in regulation of body temperature during heat stress (August). Data are least‐squares means ± SEM for vaginal temperature recorded at 15 min intervals in heifers (A), and rectal temperature (B), skin temperature (C), respiration rate (D) measured in the afternoon for bulls and heifers.

Effects of inheritance of the gene edit on the regulation of body temperature were measured during August, 2022 when heat stress was prevalent. The mean and maximum dry bulb temperatures were 26.6°C and 35.6°C and the average and maximum temperature‐humidity index, a measure of the degree of heat stress, were 77.8 and 81.0, respectively. Vaginal temperature was measured in females for two‐5‐day periods at 15‐min intervals. As shown in Figure 2A, vaginal temperature was affected by the interaction between genotype and time of day (*p* = 0.0005). Vaginal temperatures were generally lower for slick than non‐slick females, but the magnitude of the difference varied with the time of day. Overall, vaginal temperature was 39.3 ± 0.2°C for the slick females and 39.6 ± 0.2°C for non‐slick females.

Also, during August, rectal and skin temperatures and respiration rates were measured in heifers and bulls in the afternoon (i.e., when dry bulb temperatures were high) on 4 different occasions when animals were processed through a chute. Both groups experienced hyperthermia (rectal temperature >38.5°C). Rectal temperature tended to be lower for slick animals for bulls but not for heifers (genotype × sex, *p* = 0.0873) (Figure 2B). There was no effect of genotype on skin temperature (Figure 2C) or respiration rate (Figure 2D).

### 3.3 Body weight

**FIGURE 3:**
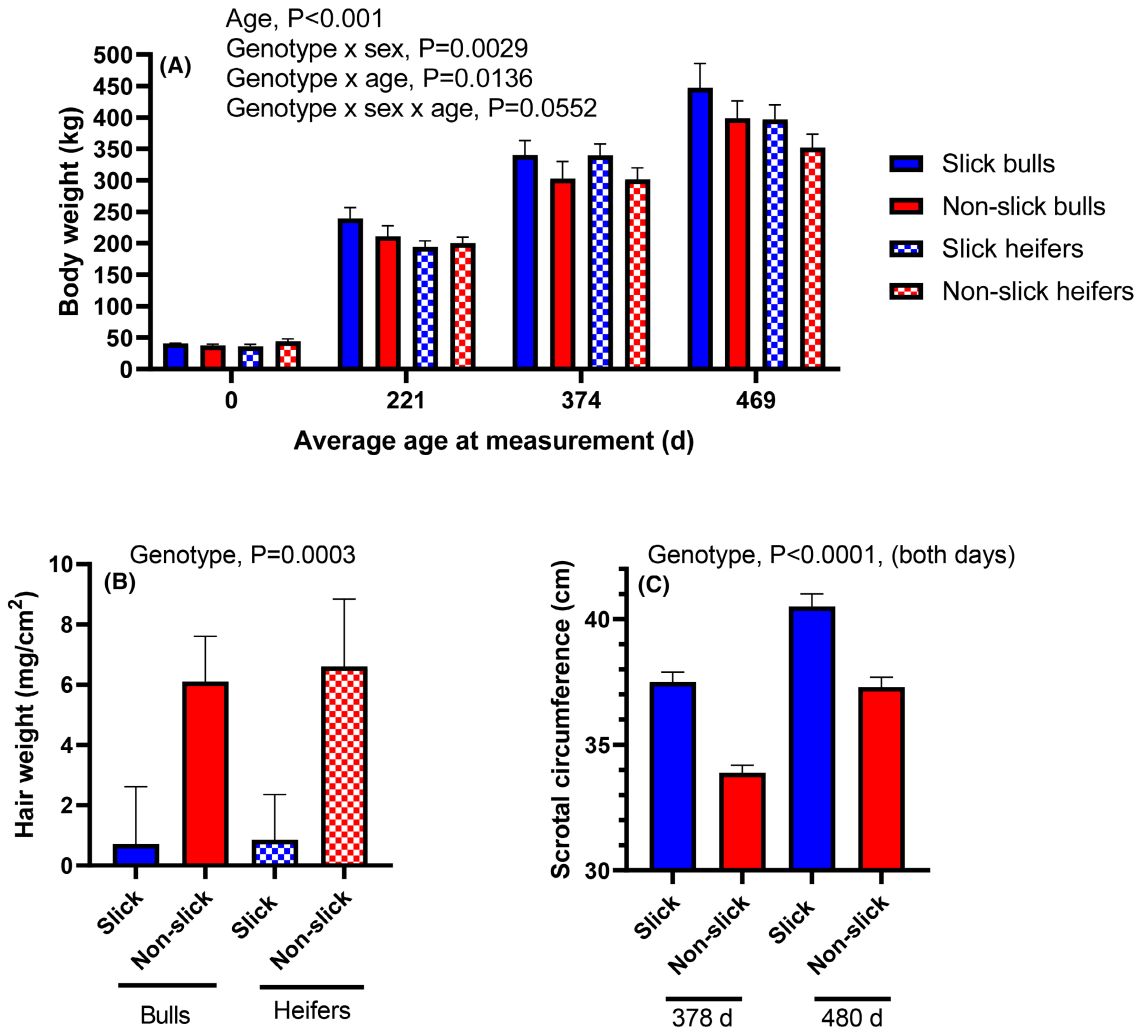
Differences between slick and non‐slick animals in body weight (A), hair weight (B) and scrotal circumference (C). Data are means ± SEM (A) or least‐squares means ± SEM.

As shown in Figure 3A, body weight was affected by genotype × sex (p = 0.0029), genotype x age (*p* = 0.0136) and genotype × sex x age (*p* = 0.0552). There was no difference in birth weight among slick and non‐slick animals but thereafter, slick animals were heavier than non‐slick animals, with differences occurring earlier in age for bulls than heifers. For example, at an average age of 221 d, slick bulls were 28 kg heavier than non‐slick bulls while slick heifers were 6 kg lighter than non‐slick heifers. At the last measurement, when animals averaged 469 d of age, the difference in body weight between slick and non‐slick animals was 49 kg for bulls and 45 kg for heifers.

### 3.4 Hair weight

Hair weight was measured on August 15 by clipping hair from a segment of the skin. Slick animals had 8.7 times less hair by weight than non‐slick animals (P = 0.0003, Figure 3B).

### 3.5 Scrotal circumference

Scrotal circumference was measured in August and November of 2022 when bulls were at an average age of 378 and 480 days (Figure 3C). Scrotal circumference was greater for slick than non‐slick bulls on both days (P < 0.0001).

### 3.6 Carcass composition

**FIGURE 4:**
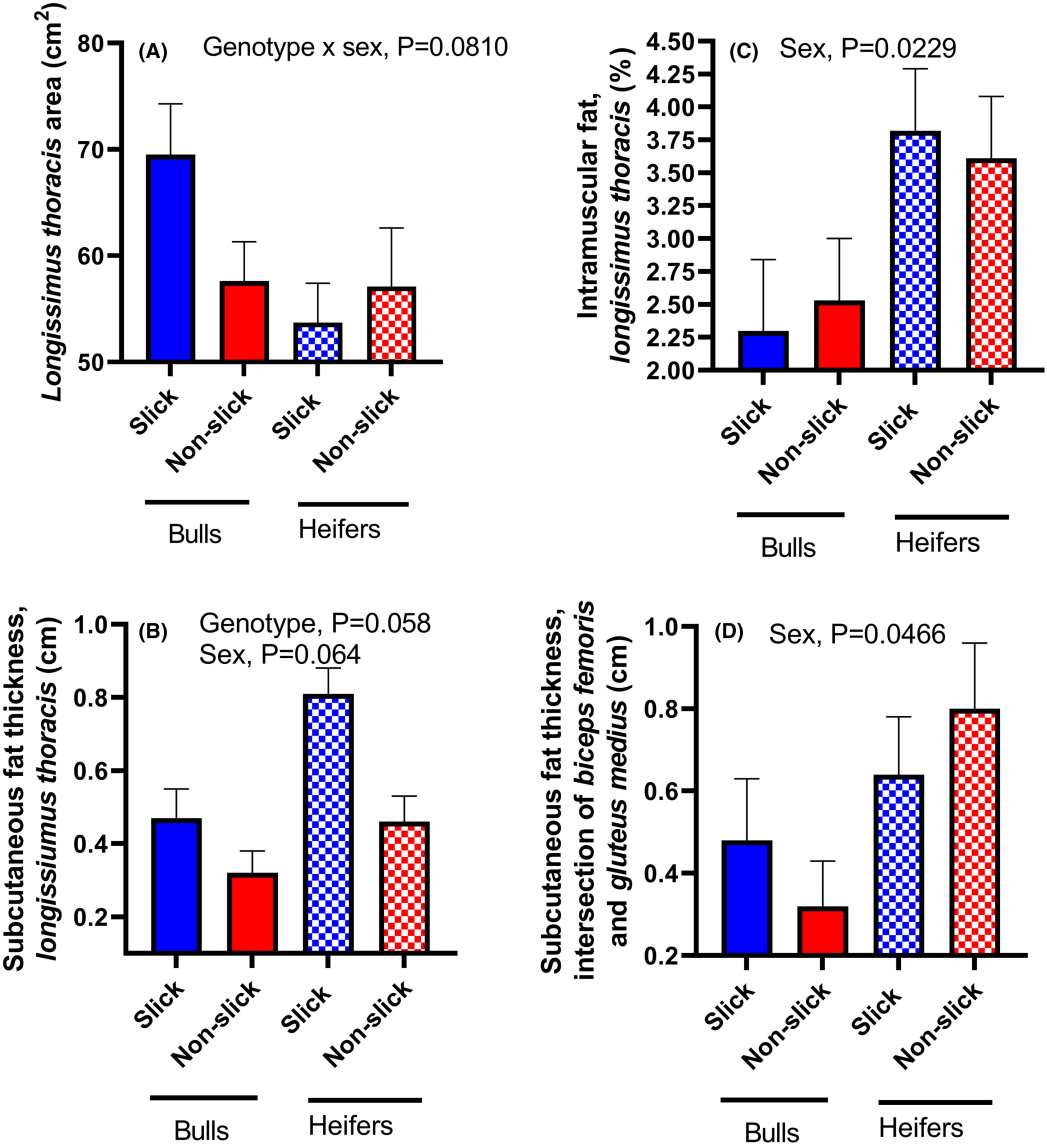
Differences between slick and non‐slick animals in carcass characteristics as determined by ultrasound at an average age of 438 days of age for slick and non‐slick animals. Data are least‐squares means ± SEM for *longissimus thoracis* cross‐sectional area (A), subcutaneous fat thickness over the *longissimus thoracis*, intramuscular fat percent in *longissimus thoracis* (C) and subcutaneous fat thickness at the intersection of the *biceps femoris* and *gluteus medius* muscles (D). [Correction added 6 August, 2024 after original online publication: In panel B, P = 0.0058 has been corrected to P = 0.058.]

Carcass composition was measured by ultrasound on November 18, 2022, after the period of heat stress was over and when animals were at an average of 469 days of age (Figure 4). The cross‐sectional area of the *longissimus thoracis* muscle tended to be larger for slick than non‐slick animals for bulls but not for heifers (genotype × sex; *p* = 0.0810, Figure 4A). Subcutaneous fat thickness over *longissimus thoracis* was also greater for slick animals (*p* = 0.0058, Figure 4B). There was no effect of genotype or the interaction of genotype with sex on intramuscular fat percent in *longissimus thoracis* (Figure 4C) or on subcutaneous fat thickness at the intersection of *biceps femoris* and *gluteus medius*. (Figure 4D). Amounts of subcutaneous fat at the latter site was less (P = 0.0466) for bulls than heifers (Figure 4D).

### 3.7 Semen characteristics

Semen collected in November and December of 2022 was evaluated for motility using computer‐assisted semen analysis and for fertilizing ability using in vitro fertilization. Results are summarized in Table 1. Ejaculate volume, sperm concentration, and total sperm per ejaculate were not affected by genotype. The same was observed for kinematic variables with one exception. The percentage of sperm exhibiting straightness was slightly lower for slick bulls (P = 0.0421). All bulls exhibited acceptable motility according to the Manual for Breeding Soundness Examination of the Society for Theriogenology.^24^ There were also no effects of genotype on the ability of sperm to fertilize oocytes (as determined by the percent of oocytes cleaving) or of the resultant embryos to develop to the blastocyst stage (Table 1).

**TABLE 1:** Semen characteristics with respect to volume, sperm concentration, kinematics, and fertilizing ability.

Variable	Slick	Non‐slick	*p*
Ejaculate volume (mL)	6.7 ± 1.6	5.9 ± 1.1	0.6514
Sperm concentration (10^6^ cells/mL)	301 ± 74	258 ± 52	0.6261
Total number of sperm/ejaculate	2127 ± 323	1686 ± 224	0.1998
Total motility (% of total)	71.3 ± 4.9	78.1 ± 3.5	0.2733
Progressive motility (% of total)	53.2 ± 5.2	62.3 ± 3.7	0.1883
Abnormal sperm (%)	27.4 ± 4.8	20.2 ± 6.0	0.2454
Average path velocity (μm/s)	99.3 ± 3.4	96.1 ± 2.3	0.4292
Curvilinear velocity (μm/s)	152.8 ± 6.8	138.9 ± 4.7	0.1302
Straight line velocity (μm/s)	90.2 ± 3.1	89.5 ± 2.2	0.8631
Linearity (%)	63.2 ± 2.2	67.4 ± 1.6	0.1584
Straightness (%)	90.8 ± 0.7	93.1 ± 0.5	0.0421
Amplitude of lateral head (μm)	5.5 ± 0.5	4.8 ± 0.3	0.2421
Beat cross frequency (Hz)	37.2 ± 1.4	38.2 ± 1.0	0.5493
Oocytes that cleaved (%)	86.1 ± 1.4	88.4 ± 1.4	0.3788
Cleaved embryos becoming blastocysts (%)	41.7 ± 2.4	45.3 ± 1.7	0.2920
Oocytes becoming blastocysts (%)	48.3 ± 2.3	51.1 ± 1.6	0.3665

### 3.8 Circulating concentrations of prolactin and IGF1

**FIGURE 5:**
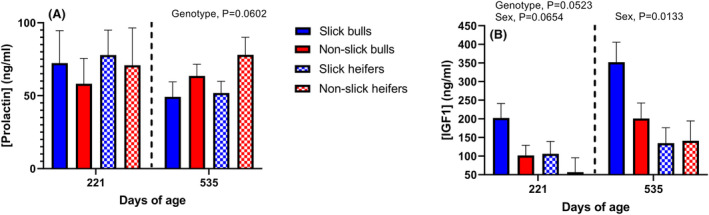
Circulating concentrations of plasma prolactin and insulin‐like growth factor 1 (IGF1). Data are least‐squares means ± SEM.

Prolactin and IGF1 concentrations in plasma were measured at weaning on March 15, 2022 (average age = 221 days) and on January 23, 2023, when animals were at an average age of 535 days. Prolactin concentrations were not affected by genotype at 221 d of age, but slick animals had lower (P = 0.0602) concentrations of prolactin compared to non‐slick animals at 535 days of age (Figure 5A). Concentrations of IGF1 were higher (*p* = 0.0523) in slick animals at weaning, but not at 535 d of age (Figure 5B).

## 4 DISCUSSION

Long hair coats inhibit the transfer of heat from the body core to the surrounding environment and cattle with long, thick hair coats have higher rectal temperatures and respiration rates during heat stress than cattle with short coats.^25^ Natural selection has resulted in several populations of cattle having genes conferring short hair coats. One example is the Brahman breed, an indicine animal in which the responsible mutations are not known.^26^ Another example is the existence of slick mutations in *PRLR* that have been identified in Criollo taurine cattle of South and Central America.^7^, ^8^, ^9^ Inheritance of even one slick allele of *PRLR* confers increased thermotolerance as indicated by regulation of body temperature and production of milk during heat stress.^10^, ^11^, ^12^, ^13^ Here we show the effectiveness of gene‐editing to introduce slick mutations into *PRLR* and produce, in a single generation, cattle with superior ability to both limit hyperthermia during heat stress and experience greater body growth. One result was that slick animals were 45 (heifers) to 49 kg (bulls) heavier on average at ~469 days of age compared to non‐slick animals, while exhibiting carcass characteristics similar or better than non‐slick animals. Thus, the introduction of the novel slick allele not only enhanced animal health by limiting hyperthermia but also improved the sustainability of beef production by enhancing growth.

Like other slick mutations in *PRLR*,^7^, ^8^, ^9^ the mutations induced by gene editing cause translation of a truncated protein due to loss of amino acids in the C‐terminus protein including tyrosine residues responsible for activation of JAK/STAT signaling.^27^ Nonetheless, slick mutations in *PRLR* should not be considered simply as loss‐of‐function mutations because some actions of prolactin, such as inhibition of hair growth,^7^ are enhanced in cattle with a slick mutation. Moreover, examination of gene expression in the liver of slick Holsteins indicated that certain actions of prolactin were likely to be enhanced in slick animals while others were inhibited.^13^ The tendency for lower concentrations of prolactin in slick animals at 535 days of age seen here could be due to enhanced negative feedback actions of prolactin on its own secretion.^28^

One possible explanation for the increased body weight of slick animals as compared to non‐slick animals is enhanced actions of prolactin on growth rate. Prolactin has been shown to increase weight gain in rats through stimulation of feed intake^29^ although similar effects were not observed in pigs.^30^ Circulating concentrations of IGF1 at weaning were also higher in slick animals. Further work to evaluate the effect of slick mutations on metabolic hormones is warranted. Another intuitive explanation for the increased growth of slick animals was that the superior ability for thermoregulation of these animals reduced the effect of heat stress on feed intake, postabsorptive metabolism, and basal metabolic rate that together compromise growth.^30^

There was no effect of *PRLR* gene edits on male reproductive function, at least based on scrotal circumference, sperm concentration, sperm kinematics, and ability of frozen–thawed sperm to fertilize oocytes using an in vitro test of fertilizing ability. Indeed, scrotal circumference was greater in slick bulls than non‐slick ones. The role of prolactin in male reproductive function is not well understood although prolactin receptors are expressed by Sertoli cells^31^ and spermatogenic cells.^32^ Hyperprolactinemia in men has been reported associated with compromised spermatogenesis.^33^ The lack of an effect of gene editing on male reproductive function is indicative that any change in prolactin signaling due to the *PRLR* mutation is not sufficient to alter reproductive function.

In conclusion, introducing novel slick mutations in *PRLR* gene‐editing in Angus and Jersey cattle improved their ability to regulate body temperature while also enhancing capacity for growth. There were no negative consequences for any measurements of male reproductive function. Thus, gene editing represents a useful strategy for rapidly introducing thermotolerance genes into cattle.

## AUTHOR CONTRIBUTIONS

PJ Hansen and TS Sonstegard conceived the experiment, CJ Cuellar coordinated collection of data and all authors participated in collection of data. Statistical analysis was performed by Cuellar and Hansen. Cuellar wrote the first draft of the paper, and all authors edited the paper and approved the final version of the manuscript.

## CONFLICT OF INTEREST STATEMENT

Authors from Acceligen and Semex have an interest in commercialization of gene‐edited livestock. There are no other conflicts.

## Supporting information


Table S1‐S2.

## Data Availability

All raw data are deposited in Dryad and made freely available (https://doi.org/10.5061/dryad.8sf7m0cwj).
