# Supplementary material for: Consequences of gene editing of PRLR on thermotolerance, growth, and male reproduction in cattle
Source: FASEB Bioadv. 2024 Jun 18;6(8):223–34. doi: 10.1096/fba.2024-00029 (PMC11301273; doi:10.1096/fba.2024-00029)
Supplement: Supplementary file 1 — Table S1‐S2. [file FBA2-6-223-s001.pdf]

Table S1. The population of animals generated following gene editing.

Breed and genotype	Bulls	Heifers	Total
Angus			
Gene-edited	2	4	6
Wild-type	3	3[a]	6
Mosaic	1	2	3<br>(4% - 29% edited)
Jersey			
Gene-edited	2	3	5
Wild-type	1	0	1
Mosaic	0	0	0

[a] One heifer was stillborn.

Table S2. Allele frequency for *PRLR*.[a]

Breed	ID	Birth date	Sex	Genotype	Allele (frequency)
Angus	S005	2021.07.04	Heifer	bi-allelic slick	1bp ins (100%)
Angus	S006	2021.07.04	Bull	wt	wild type
Angus	S007	2021.07.07	Bull	bi-allelic slick	10bp del (53%)<br>4bp ins (47%)
Angus	S008	2021.07.15	Bull	Mosaic	wild type (90%)<br>1bp ins (9%)<br>1bp del (1%)
Angus	S009	2021.07.23	Heifer	Mosaic	wild type (96%)<br>7bp del (4%)
Angus	S010	2021.07.26	Bull	wt	wild type
Angus	S011	2021.07.26	Bull	wt	wild type
Angus	S012	2021.07.26	Heifer	bi-allelic slick	95bp del (59%)<br>92bp del (23%)<br>34bp del (18%)
Angus	S013	2021.07.28	Bull	bi-allelic slick	14bp del (51%)<br>10bp del (49%)
Angus	S014	2021.08.05	Heifer	wt	wild type
Angus	S015	2021.08.07	Heifer	wt	wild type
Angus	S016	2021.08.11	Heifer	Mosaic	wild type (71%)<br>10bp del (29%)
Angus	S017	2021.08.10	Heifer	wt	wild type
Jersey	S018	2021.09.12	Bull	bi-allelic slick	10bp del (50%)<br>7bp del (50%)

Angus	S019	2021.09.15	Heifer	bi-allelic slick	215bp del (41%)<br>195bp del (41%)<br>14bp del (9%)<br>4bp del (9%)
Angus	S020	2021.09.15	Heifer	bi-allelic slick	10bp del (53%)<br>1bp del (47%)
Jersey	S021	2021.09.15	Bull	wt	wild type
Jersey	S022	2021.09.16	Heifer	bi-allelic slick	110bp del (75%)<br>7bp ins (25%)
Jersey	S023	2021.09.16	Heifer	bi-allelic slick	10bp del (51%)<br>10bp del* (49%)
Jersey	S024	2021.09.17	Heifer	bi-allelic slick	128bp del; 7bp ins (67%)<br>10bp del (33%)
Jersey	S025	2021.09.18	Bull	bi-allelic slick	55bp del (66%)<br>5bp ins (33%)

[a] Abbreviations: del, deletion; ID, identification; ins, insert; wt, wild-type
